# Google as a cancer control tool in Queensland

**DOI:** 10.1186/s12885-017-3828-x

**Published:** 2017-12-04

**Authors:** Xiaodong Huang, Peter Baade, Danny R. Youlden, Philippa H. Youl, Wenbiao Hu, Michael G. Kimlin

**Affiliations:** 1 Faculty of Science, Health, Education and Engineering, University of the Sunshine Coast, Sippy Downs Dr, Sippy Downs, QLD 4556 Australia; 2 Cancer Council Queensland, Brisbane, QLD Australia; 3 School of Public Health and Social Work, Institute of Health and Biomedical Innovation, Queensland University of Technology, Brisbane, QLD Australia; 4 Menzies Health Institute Queensland, Griffith University, Gold Coast, QLD Australia

**Keywords:** Google Trends, Search volume indexes, Cancer incidence, Age standardised rates, Cross-correlation

## Abstract

**Background:**

Recent advances in methodologies utilizing “big data” have allowed researchers to investigate the use of common internet search engines as a real time tool to track disease. Little is known about its utility with tracking cancer incidence. This study aims to investigate the potential correlates of monthly internet search volume indexes (SVIs) and observed monthly age standardised incidence rates (ASRs) for breast cancer, colorectal cancer, melanoma and prostate cancer.

**Methods:**

The monthly ASRs for the four cancers in Queensland were calculated using data from the Queensland Cancer Registry between January 2006 and December 2012. The monthly SVIs of the respective cancer search terms in Queensland were accessed from Google Trends for the same period. A time series seasonal decomposition method was performed to detect the seasonal patterns of SVIs and ASRs. Pearson’s correlation coefficient and time series cross-correlation analysis were used to assess the associations between SVIs and ASRs. Linear regression models were used to examine the power of SVIs to predict monthly in ASRs.

**Results:**

Increases in the monthly ASRs of the four cancers were significantly correlated with increases in the monthly SVIs of the respective cancers except for colorectal cancer. The predictive power of the SVIs to explain variances in the corresponding ASRs varied by cancer type, with the percent explained ranging from 5.6% for breast cancer to 17.9% for skin cancer (SVI) with melanoma (ASR). Some improvement in the variation explained was obtained by including more search terms or lagged SVIs for the respective cancers in the linear regression models. The seasonal analysis indicated that the SVIs peaked periodically at around their respective cancer awareness months.

**Conclusions:**

Using SVIs from a popular internet search engine was only able to explain a small portion of changes in the respective ASRs. While an expanded regression model explained a higher proportion of variability, the interpretation of this was difficult. Further development and refinement of this approach will be needed before search-based cancer surveillance can provide useful information regarding resource deployment to guide cancer control and track the impact of cancer awareness and education programmes.

## Background

Online search engines provide an opportunity for people to search and obtain health information about diagnosis, symptoms and treatments for most diseases. Online health data have been recently considered as a potential and valuable source of developing surveillance systems of disease and establishing early warning systems of emerging infectious disease [1–3]. Researchers therefore have been seeking suitable methods to develop efficient tools for disease surveillance systems using internet search queries [4–9].

In Queensland, an increasing number of new cancer cases is mostly due to population growth and ageing. In total, 26,335 people were diagnosed with cancer during 2013 in Queensland [10]. The four most common cancers diagnosed were prostate cancer, breast cancer, melanoma and colorectal cancer [11, 12].

Cancer-related internet searches are one of the most common search behaviours for people seeking health information. Around 35% of online health seekers were estimated to have used the internet for the purpose of self-diagnosis [13]. A recent study found that internet searching was a very important source of health information about treatment for cancer patients among 77 German websites [14]. Previous studies have shown between 39% to 60% of cancer patients are using the internet for health related resources [15] and up to a further 20% of cancer patients in the developed world access the internet with the assistance of family and friends [16]. Online health information has the potential to assist individuals and their families in making treatment-related decisions [17, 18].

Google is the most popular search engine and accounts for approximately 90% of all online searches in Australia [19]. Thousands of daily online health-related search queries provide information on collective health trends and are recorded through information repositories such as Google Trends [4, 20]. Google Trends stores large populations of internet search queries and is a potential option for a robust and real-time surveillance system of epidemics and diseases [21]. Seasonal public health messages, such as those for melanoma and skin cancer prevention, may be able to be assessed for effectiveness using such data. As such, this study aims to investigate the association between observed age standardised incidence rates (ASRs) for common types of cancer and measures of internet cancer-search queries reported by Google Trends to explore whether the internet search activity can support cancer surveillance and guide future prevention and support programs.

## Methods

### Data collection

De-identified unit record data on all persons diagnosed with breast cancer, colorectal cancer, melanoma and prostate cancer in Queensland between January 2006 and December 2012 were obtained from the Queensland Cancer Registry. The Queensland Cancer Registry supplies Cancer Council Queensland with de-identified unit record data for research purposes on an annual basis under a Memorandum of Understanding with the data custodian (Queensland Health). As only de-identified information was used, specific ethics approval was not required for this study. Population data are publicly available from the Australian Bureau of Statistics website.

Mid-year estimated resident population data stratified by sex and 5-year age groups were sourced from the Australian Bureau of Statistics. In the absence of monthly population estimates, the person-years at risk in each month was approximated from the yearly population data according to the number of days in each month.

Google Trends provides a percentage of all searches (e.g., each point data is divided by the total searchers of the geography) for a particular search term within a given time period and location [22]. We obtained search volume indexes (SVIs) for individual search terms through Google Trends which provides normalised monthly time series data with indexes between 0 and 100. Google calculated the monthly SVIs by analysing a fraction of the total cancer-related searches during 1 month within a certain location in Google Web [21]. While colorectal cancer (also referred to as bowel cancer) comprises both colon cancer and rectal cancer, the monthly SVI for “rectal cancer” was not available for Queensland in Google Trends. The search term “skin cancer” was also included in the analysis although it includes melanoma, keratinocyte cancers and other skin cancers. Therefore, the specific search terms investigated in the study included breast cancer, colorectal cancer, bowel cancer, colon cancer, melanoma, skin cancer and prostate cancer. The monthly SVIs of all search terms for Queensland were extracted from the SVI graphs which were provided by Google Trends Web between January 2006 and December 2012, corresponding to the same period as the cancer incidence data.

### Statistical analysis

ASRs were calculated by directly standardising the age-specific incidence rates using the 2001 Australian standard population. Pearson correlation (2-tailed test) was used to quantify the linear relationships between the monthly ASRs and the monthly SVIs. Time series cross-correlation functions (CCFs) were used to examine the lagged relationships, that is, whether the monthly ASRs in Queensland are correlated with the SVIs of preceding months. The simple linear regression models were used to estimate the predictive power of the monthly SVIs for explaining the variances in the monthly ASRs of the respective cancers. The multiple linear regression model was also used to examine the predictive power of the combination effects of the multiple SVIs and the lagged SVIs on the changes in the respective ASRs. The multiple linear regression model is given by:

$$ \ln (y)={\beta}_0+{\beta}_1\ln \left({x}_1\right)+\dots +{\beta}_{\mathrm{m}}\mathit{\ln}\left({x}_{\mathrm{m}}\right)+{\varphi}_1\ln \left({l}_1\right)+\dots +{\upvarphi}_{\mathrm{n}}\mathit{\ln}\ \left({l}_{\mathrm{n}}\right) $$

where *y* is the monthly ASRs for the four cancers; *x* represents the monthly SVIs for the respective cancers; *l* is the lagged SVIs for the respective cancers based on the lagged relationships from the CCFs. *β* and *φ* are regression coefficients. Both the monthly ASRs and the monthly SVIs were log-transformed to increase stationarity of the time series in correlation analysis and linear regression models.

A time series seasonal decomposition method was used to detect the periodicity effects of both the monthly time series data on SVI and ASR. To explore the impact of seasonality and trends, we decomposed the SVIs and ASRs into the seasonal and trend series [23]. We adopted the model: *Y*_*t*_ *= T*_*t*_ *+ S*_*t*_ *+ C*_*t*_ *+ E*_*t*_. Here *Y*_*t*_ denotes the original times series of either the SVIs or ASRs; *T*_*t*_, *S*_*t*_, *C*_*t*_ and *E*_*t*_ denote the trend component, the seasonal component (seasonal factor), the cycle component (seasonal adjusted time series) and the residual component, respectively.

## Results

**Fig. 1:**
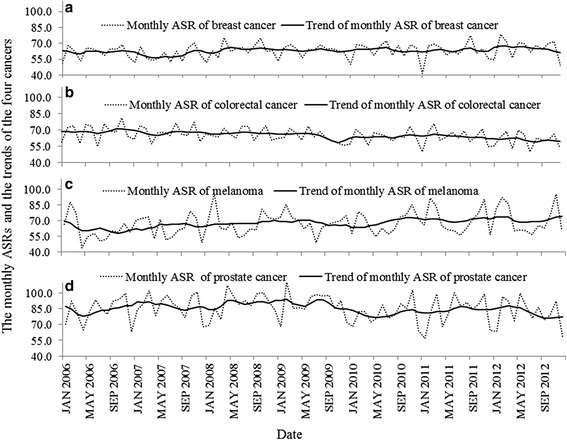
Plot of the monthly ASRs and the monthly ASR trends of the four cancers ((**a**) breast cancer, (**b**) colorectal cancer, (**c**) melanoma and (**d**) prostate cancer) over the study period in Queensland

Table 1 shows the summary statistics for the monthly incidence ASRs of breast cancer, colorectal cancer, melanoma and prostate cancer in Queensland. Prostate cancer had the highest mean ASR (85.3 per 100,000 population) and breast cancer had the lowest mean ASR (63.0 per 100,000 population). Figure 1 shows the temporal variability of the monthly ASRs and the ASR trends (*T*_*t*_) for the four cancers over the study period.

**Table 1:** Summary statistics of observed monthly ASRs (per 100,000 population) for the four cancers, Queensland, 2006-2012

Cancer	Mean	STD	Minimum	Maximum	Percentiles		
					25	50	75
Breast	63.0	6.8	41.3	78.3	58.7	64.3	67.8
Prostate	85.3	12.0	56.8	110.3	76.4	86.2	93.7
Melanoma	67.6	11.4	44.0	95.8	60.4	65.8	73.8
Colorectal	65.3	6.6	50.0	81.2	61.0	65.1	69.7

Table 2 shows summary statistics for the monthly SVIs of all search terms for Queensland. The highest mean monthly SVI among all search terms was generated by “prostate cancer” (mean SVI = 57.7), followed by “bowel cancer” (mean SVI = 46.7) and “melanoma” (mean SVI = 43.8). The lowest mean monthly SVIs were generated by the search terms “colorectal cancer” (mean SVI = 13.7), “colon cancer” (mean SVI = 19.6) and “breast cancer” (mean SVI = 25.3).

**Table 2:** Summary statistics of Google Trends Queensland monthly SVIs for all search terms, 2006-2012

Search terms	Mean	STD	Minimum	Maximum	Percentiles		
					25	50	75
Breast cancer	25.3	11.4	13.0	100.0	19.3	23.0	27.0
Prostate cancer	57.7	13.7	29.0	100.0	48.0	57.0	65.8
Melanoma	43.8	11.1	23.0	69.0	36.3	43.0	50.8
Skin cancer	35.4	10.9	22.0	75.0	27.3	33.0	39.0
Bowel cancer	46.7	14.6	24.0	100.0	37.0	43.0	55.8
Colon cancer	19.6	5.9	7.0	42.0	16.0	19.0	23.0
Colorectal cancer	13.7	5.8	6.0	34.0	10.0	13.0	16.8

**Fig. 2:**
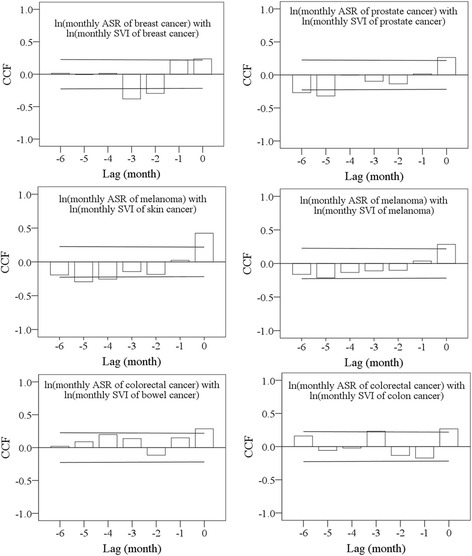
Cross-correlation functions between log-transformed monthly ASRs and log-transformed monthly SVIs of the respective search terms for the four cancers. The two solid lines are critical values for cross-correlation (at the 5% level)

Pearson correlation coefficients showed that log-transformed monthly SVIs of all search terms (excluding “colorectal cancer”) were significantly associated with the respective log-transformed monthly ASRs. The highest correlation was between the log-transformed monthly SVI of the search terms “skin cancer” and the log-transformed monthly ASR of melanoma (*r* = 0.424, *P* < 0.000) (Table 3). The CCFs (Fig. 2) showed the cross correlations with negative lags between the log-transformed monthly ASRs and the log-transformed monthly SVIs. In Fig. 2, a negative lag indicates that the SVI has been measured before the month the cancer is clinical diagnosis confirmed in. A negative cross correlation indicates that increase in the SVI is associated with decrease in the ASR. The monthly ASRs for the four cancers were significantly and positively correlated with the respective SVIs at lag 0 months. There were significant negative correlations at negative lags of 2-3 months between the ASR and the SVI for breast cancer. Similarly, there were significant negative correlations at negative lags of 4-5 months between the ASR of melanoma and the SVI of skin cancer. Moreover, there were also significant negative correlations at negative lags of 5-6 months between the ASR and the SVI for prostate cancer.

**Table 3:** Pearson’s correlation coefficients between log-transformed SVIs of search terms and log-transformed ASRs of the four cancers

Log-transformed ASR	Log-transformed SVI	Pearson correlation coefficient	*P*-value
Melanoma	Melanoma	0.284	0.009
Melanoma	Skin cancer	0.424	0.000
Breast cancer	Breast cancer	0.236	0.030
Prostate cancer	Prostate cancer	0.264	0.015
Colorectal cancer	Bowel cancer	0.287	0.008
Colorectal cancer	Colon cancer	0.268	0.014
Colorectal cancer	Colorectal cancer	0.110	0.321

**Fig. 3:**
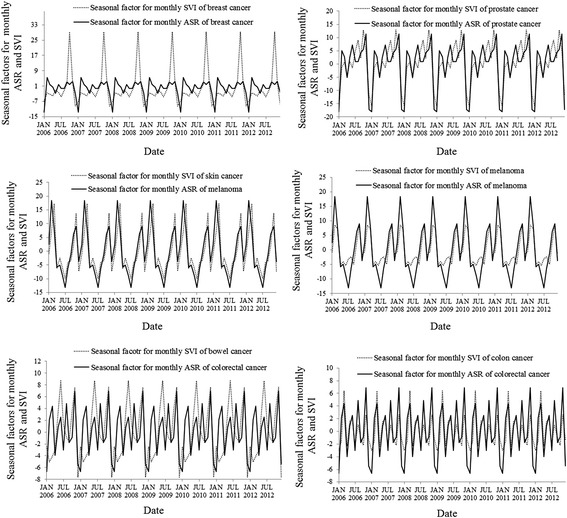
The patterns of seasonal factors for log-transformed monthly ASRs and log-transformed monthly SVIs of the respective search terms for the four cancers

The time series seasonal decomposition analysis found similar patterns of the seasonal factors (*S*_*t*_) between the monthly SVIs of the search terms “prostate cancer”, “skin cancer”, “melanoma”, “bowel cancer” and “colon cancer” and the respective monthly ASRs. However, the seasonal patterns for SVI for breast cancer were different to those of the ASR (Fig. 3). The lowest values of the seasonal factors for the monthly SVIs and the monthly ASRs of breast cancer, bowel cancer, colorectal cancer, colon cancer and prostate cancer were detected yearly in each December or January, and in June or July for melanoma and skin cancer. The highest values of seasonal factors for the monthly SVIs of the search terms “breast cancer”, “bowel cancer”, “colon cancer”, “melanoma” “skin cancer” and “prostate cancer” usually presented in October, June, May, February, March and October of each year, respectively. However, the highest values of seasonal factors for the monthly ASRs occurred in each February for breast cancer and melanoma and exhibited in each November for colorectal cancer and prostate cancer (Fig. 3).

**Fig. 4:**
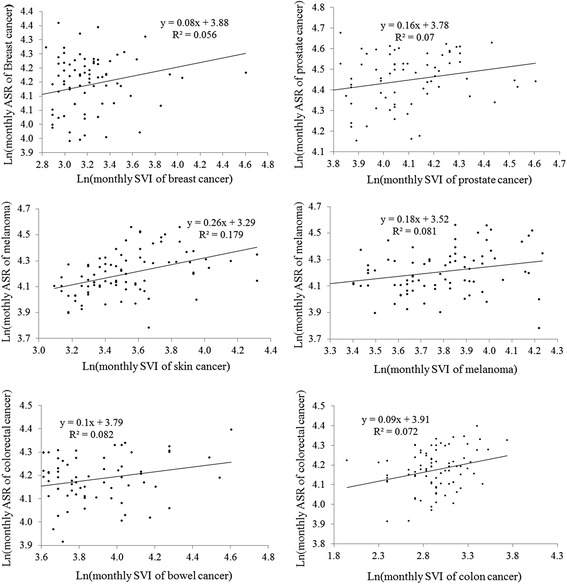
The scatter plots with the fitted linear lines and the simple linear regression models between log-transformed monthly ASRs and log-transformed monthly SVIs of the respective search terms

Figure 4 shows the scatter plots with the fitted simple linear regression lines and the simple linear regression models between the log-transformed monthly ASRs and the log-transformed monthly SVIs. All log-transformed monthly SVIs of the search terms, except for the search term “colorectal cancer”, were significantly positively associated with the respective monthly ASRs in the simple linear regression models. Among the four cancers, the log-transformed monthly SVI of skin cancer explained the highest percentage (*R* ^2^ = 17.9%) of the total variance in the log-transformed monthly ASR of melanoma, whereas the SVI of breast cancer explained the lowest percentage (5.6%) of the total variance in the monthly ASR (Fig. 4).

The multiple linear regression models for the four cancers indicated that including multiple search terms and lagged SVIs could explain a higher percentage of the total variance in the respective ASRs compared with using a single predictor (Table 4 and Fig. 4). The highest percentage (R^2^ = 39.5%) of total variance in the monthly ASR of melanoma could be explained by the respective monthly SVIs and the average of monthly SVIs of skin cancer at lags of 4 and 5 months.

**Table 4:** The multiple linear regression models for the four cancers

Model	Response variable	Predictor	Coefficient	*P*-value	R^2^
1	Ln(monthly ASR of breast cancer)	Intercept	4.47	<0.000	0.206
		Ln(monthly SVI)	0.057	0.098	
		Average of monthly SVIs at lags of 2 and 3 months	−0.162	0.0002	
2	Ln(monthly ASR of prostate cancer)	Intercept	5.054	<0.000	0.225
		Ln(monthly SVI)	0.151	0.017	
		Average of monthly SVIs at lags of 5 and 6 months	−0.302	0.0004	
3	Ln(monthly ASR of melanoma)	Intercept	3.66	<0.000	0.395
		Ln(monthly SVI of melanoma)	0.161	0.016	
		Ln(monthly SVI of skin cancer)	0.231	0.0003	
		Average of monthly SVIs of skin cancer at lags of 4 and 5 months	−0.245	0.0004	
4	Ln(monthly ASR of colorectal cancer)	Intercept	3.629	<0.000	0.129
		Ln(monthly SVI of colorectal cancer)	0.002	0.935	
		Ln(monthly SVI of bowel cancer)	0.086	0.032	
		Ln(monthly SVI of colon cancer)	0.072	0.054	

## Discussion

While the study revealed some associations between the monthly ASRs of the four cancers and the respective monthly SVIs, there was wide variability in the explanatory power of the monthly SVIs across the cancer types in relation to the respective monthly ASRs. The implications for each type of cancer will therefore be considered individually. There was some improvement in prediction of the ASRs by including more search terms or lagged SVIs in the multiple linear regression models in the study.

### Breast cancer

There was a significant correlation between the monthly ASR of breast cancer and the respective SVI in the study. Although the increase in the monthly ASR followed the increase in monthly SVI, the monthly ASR was also significantly negatively correlated with the monthly SVI at lags of 2-3 months before diagnosis. Australia offers free screening mammograms for women aged 40 years and over every 2 years. Some women will be asked to come back for more tests if anything suspicious is found on the initial mammogram. The negative lagged relationship between the ASR and SVI of breast cancer could be explained by women conducting prior online searches while waiting for a confirmed diagnosis. However, most women (more than 95% of cases) do not have breast cancer after they receive more tests [24, 25]. Here, one possible explanation for the negative correlation between the lagged SVI and the ASR for breast cancer could be prior online search activities, amplified by the people who were suspected to have breast cancer or the person who only want to understand the knowledge of breast cancer, however many other factors may be contributing to this association.

### Prostate cancer

A current increase in the monthly ASR of prostate cancer was significantly related to a current increase in the respective monthly SVI in the study. Additionally, the monthly SVI was also significantly negatively correlated with the monthly ASR and shifted forward 5-6 months. Early detection of prostate cancer is complex, with the PSA test being unable to differentiate between life threatening prostate cancers that spread rapidly and those slow growing prostate cancers that require no treatment or intervention. The symptoms of prostate cancer are also similar to other benign prostatic conditions. The current early detection of prostate cancer is difficult and prostate cancer often develops slowly without demanding treatment and affecting patients’ lifestyles [26]. It is therefore possible that cancer patients recently diagnosed with prostate cancer might be caught unaware and require more immediate cancer information because of the lack of any prior symptoms. On the other hand, increase in prior online search activities of the preceding 5-6 months was negatively associated with the monthly ASR, which could be indicated that most online search activities might be executed by people without prostate cancer.

### Melanoma

An increase in the monthly ASR of melanoma was significantly related to a simultaneous increase in the monthly SVI of both skin cancer and melanoma. Moreover, the monthly ASR was significantly negatively related to the monthly SVI of skin cancer at lags of 4-5 months. Melanoma often is related to changes in asymmetry, irregular border and uneven colour of skin lesions [27]. It is possible that significant changes in the skin by visual observation may lead people to seek information about skin cancer before diagnosis, whereas patients would urgently need cancer information at the time of diagnosis. Hence, the negative relationship between the ASR and the lagged SVIs might be explained that the online search activities from non-patients amplified the SVI. Additionally, it is interesting that the lowest values of seasonal factors occurred during the Australian winter (in each June for the monthly SVI of melanoma and in each July for both the monthly SVI of skin cancer and the monthly ASR of melanoma), and that the periodical peak ASR was observed in each February in the summer in Australia, which tend to be the coldest or hottest weather conditions, respectively. A previous study also found increased SVI of melanoma during the summer season in US [28]. Finally, we found that the SVI of skin cancer explained the highest percentage of the total variance in the monthly ASR of melanoma compared to the other cancers in the simple linear regression model. Of interest is that the term “skin cancer” encompasses both melanoma and non-melanoma skin cancers. It might reflect that online users (patients and non-patients alike) could not clearly distinguish the difference between melanoma and non-melanoma skin cancer, which resulted in “skin cancer” as a better predictor of the ASR of melanoma than the search term “melanoma”.

### Colorectal cancer

The monthly ASR of colorectal cancer was significantly positively associated with the monthly SVIs of bowel cancer and colon cancer but not with the monthly SVI of colorectal cancer. Positive significant correlations indicated that an increase in ASR led to an increase in the respective SVI. Generally, bowel cancer often grows without any initial symptoms [29]. However, the National Bowel Cancer Screening Program provides a free test for Australians aged over 50 to detect bowel cancer in its early stages [30]. It is not surprising that an increase in the ASR might lead to an increase in the SVI due to more online searches by newly diagnosed patients. Moreover, the mean value of the SVI of bowel cancer was greater than that of the SVI of colon cancer, which highlights an important message about communication to the general public – bowel cancer is a much more common term than colorectal cancer.

### Seasonality

The seasonal decomposition analysis revealed that both the monthly ASRs and the monthly SVIs, except melanoma, had the lowest values in each December or January, while the seasonal factor of the monthly SVI of skin cancer showed the second lowest values in each December. It might be explained that the Christmas/New Year holiday period resulted in decreased numbers of people seeking medical diagnosis [31], possibly due to fewer specialists being available at this time, competing priorities for potential patients who may be also less likely to search for online health information relating to cancer symptoms. In addition, we found that the periodical peak SVIs of bowel cancer (June), breast cancer (October) and the periodical second peak SVIs of melanoma and skin cancer (November) corresponded to the respective awareness months for those cancers in Australia, while the periodical peak SVI of prostate cancer occurred in each October and lagged 1 month behind the international prostate cancer awareness month (September). Commonly, the specific cancer awareness months are held every year to raise awareness and as an avenue for fund-raising [32–35]. The present results might reflect that increased awareness of cancer patients leads to seeking further information about cancer. Our findings suggest that the frequency of online search activity could be prompted by cancer awareness events, which has been found in previous studies [18, 36]. Cancer prevention agencies and health professionals should therefore continue to integrate the dissemination of up-to-date and targeted information through Internet websites into broader cancer awareness events.

Important limitations of this study should be acknowledged. First, we are using ecological data to measure a complex association, in particularly the lack of information on the individual-level link between cancer diagnosis and internet searches limits our ability to make any definitive conclusions. Second, this study only focused on search terms for the specific cancers, rather than searches which involved relevant symptoms or treatments. Third, the Google Trends SVIs could not indicate how many searchers were cancer patients among the total searchers on Google Web. Fourth, although Google is the most popular search engine in Australia, other search engines and specific health Web sites are still available to internet users searching for cancer information. Using a combination of search engines could improve the understanding of the current cancer situation in Australia. In addition, official monthly population data was not available for use in the denominators for the rate calculations, and so this data had to be approximated from annual data.

## Conclusions

We found only some evidence of a small association between online cancer-seeking information behaviours and the incidence of some types of cancer in Queensland, with a peak in search activities consistent with the annual cancer-specific awareness campaigns in this country. While an expanded regression model explained a higher proportion of variability, the interpretation of this was difficult. While search-based cancer surveillance has the potential to provide useful information regarding resource deployment to guide cancer control and track the impact of cancer awareness and education programmes, at best, these types of big data information are able to supplement the standard data collected in population-based cancer registries, rather than be a substitute for them. However, further development and refinement of the methodological approach and data availability will be needed before it provides useful insights into the burden of cancer in this country.
